# Kangfuxin solution perianal warm moist compress combined with music therapy for anal fullness after endoscopic treatment of internal hemorrhoids

**DOI:** 10.3389/fmed.2026.1835242

**Published:** 2026-05-11

**Authors:** Fen Gao, Fan Zhang, Yan Cao, Xiaoyun Fu, Jiangang Wang, Qin Liu

**Affiliations:** 1 Department of Gastroenterology, Yunnan Third People's Hospital, Kunming, Yunnan, China; 2 School of Nursing, Yunnan University of Traditional Chinese Medicine, Kunming, Yunnan, China

**Keywords:** anal fullness, factorial design, internal hemorrhoids, Kangfuxin solution, music therapy, postoperative nursing

## Abstract

**Background:**

Anal fullness commonly occurs following endoscopic treatment of internal hemorrhoids, yet targeted nursing interventions remain limited. This study evaluated the individual and combined effects of Kangfuxin solution (*Periplaneta americana* extract) perianal warm moist compress and music therapy on this symptom.

**Methods:**

In this prospective 2 × 2 factorial-design study, 120 patients with Grade II/III internal hemorrhoids undergoing endoscopic treatment were randomized (*n* = 30 per group): Kangfuxin compress plus music therapy (A), Kangfuxin compress alone (B), music therapy alone (C), and routine care (D). Interventions were administered twice daily for 5 days. Outcomes included anal fullness (Visual Analogue Scale [VAS]), symptom duration, quality of life (WHOQOL-BREF), and therapeutic efficacy. Factorial main effects and interaction were assessed using two-way ANOVA.

**Results:**

Group A showed significantly lower VAS scores at all postoperative time points (all *p* < 0.05), reaching 0.82 ± 0.53 by day 7 versus 2.53 ± 0.88 in Group D. Generalized estimating equations confirmed a significant time-by-intervention interaction (*p* < 0.001). Two-way factorial ANOVA revealed significant main effects for both the Kangfuxin solution factor (*F* = 42.15, *p* < 0.001) and the music therapy factor (*F* = 15.83, *p* < 0.001), with a non-significant interaction (*F* = 0.42, *p* = 0.518), indicating additive rather than synergistic effects. Symptom duration was shortest in Group A (3.85 ± 1.12 days) versus Groups B (4.76 ± 1.25), C (5.92 ± 1.38), and D (7.21 ± 1.56; all *p* < 0.05). Group A achieved the highest WHOQOL-BREF scores and total effective rate (96.67% vs. 70.00%). Intervention method was the strongest predictor of quality of life (*β* = 0.682, *p* < 0.001). Adverse events were limited to mild skin reactions (2.5%).

**Conclusion:**

Kangfuxin solution perianal warm moist compress combined with music therapy effectively alleviates anal fullness, shortens symptom duration, and improves quality of life after endoscopic hemorrhoid treatment. The two interventions exert additive benefits, and their combination outperforms either modality alone. This safe multimodal intervention warrants integration into postoperative nursing protocols.

## Introduction

Hemorrhoidal disease is one of the most prevalent anorectal conditions, significantly impairing patients’ quality of life (1). Internal hemorrhoids represent the most common subtype, with clinical manifestations including rectal bleeding, prolapse, and anal fullness (2). Anal fullness in the context of hemorrhoidal disease refers to a subjective sensation of rectal pressure, heaviness, or distension in the perianal region, which may be accompanied by a feeling of incomplete evacuation or a persistent urge to defecate (3). This symptom is distinct from pain per se, although the two frequently coexist, and is thought to arise from tissue edema, venous congestion, and local inflammatory responses following anorectal procedures (2). In recent years, advances in minimally invasive surgical concepts and the growing emphasis on enhanced postoperative recovery have driven the development of novel treatment modalities for internal hemorrhoids (4). Endoscopic approaches, including rubber band ligation (RBL) and injection sclerotherapy (IS), have emerged as important therapeutic options, offering advantages such as reduced tissue trauma, decreased intraoperative bleeding, and accelerated recovery compared with conventional surgical techniques (4–6).

Despite these benefits, postoperative anal fullness remains a common and distressing complication that causes considerable discomfort and may impede recovery (2). Effective nursing interventions targeting this symptom are therefore of considerable clinical importance. Kangfuxin solution, an extract derived from *Periplaneta americana* (American cockroach), is a traditional Chinese medicine preparation with established anti-inflammatory, wound-healing, and tissue-regenerating properties (7, 8). When administered as a perianal warm moist compress, Kangfuxin solution enables direct topical delivery of its bioactive components to the affected tissue, while the thermal application enhances local blood circulation and promotes tissue repair (7, 9). Concurrently, music therapy has gained recognition as a non-pharmacological intervention that modulates physiological and psychological responses through auditory stimulation, with demonstrated efficacy in reducing postoperative pain and anxiety (10, 11).

Although both Kangfuxin solution warm moist compress and music therapy have individually shown promise in postoperative anorectal care, evidence regarding their combined application specifically for anal fullness following endoscopic treatment of internal hemorrhoids remains limited. This study aimed to evaluate the independent and combined effects of Kangfuxin solution perianal warm moist compress and music therapy on anal fullness symptoms, symptom duration, quality of life, and therapeutic efficacy in patients undergoing endoscopic treatment for internal hemorrhoids, with the goal of providing evidence-based guidance for clinical nursing practice.

## Methods

### Study design

This prospective, single-center, four-arm, parallel-group comparative study employed a 2 × 2 factorial design to evaluate the independent and combined effects of Kangfuxin solution perianal warm moist compress and music therapy on anal fullness following endoscopic treatment of internal hemorrhoids. Patients were allocated to one of four groups: Group A (Kangfuxin solution perianal warm moist compress combined with music therapy), Group B (Kangfuxin solution perianal warm moist compress alone), Group C (music therapy alone), and Group D (routine postoperative nursing care). The factorial design enabled simultaneous assessment of each intervention’s individual contribution and their potential interaction, while all groups received standardized routine postoperative care as the baseline nursing protocol.

The randomization sequence was generated by an independent statistician using a computer-generated block randomization scheme (block size of 4) stratified by hemorrhoid grade (Grade II vs. Grade III). Group assignments were concealed in sequentially numbered, sealed opaque envelopes prepared by administrative staff who were not involved in patient care, intervention delivery, or outcome assessment. Envelopes were opened in strict sequential order only at the time of enrollment, after the patient had provided written informed consent and confirmed all eligibility criteria. The research nurse who performed the allocation was not involved in the delivery of interventions, outcome assessment, or data analysis, ensuring adequate separation of roles to minimize the risk of selection bias.

### Participants

A total of 120 patients who underwent endoscopic minimally invasive treatment for internal hemorrhoids in the Department of Gastroenterology at the Third People’s Hospital of Yunnan Province between June 2023 and May 2024 were enrolled. All patients were diagnosed with Grade II or Grade III internal hemorrhoids based on the Goligher classification system, confirmed through clinical examination and anoscopy. Eligible patients underwent endoscopic rubber band ligation (RBL), endoscopic injection sclerotherapy (IS), or a combination of both modalities, as determined by the treating physician based on hemorrhoid grade and clinical presentation. This study was approved by the Institutional Ethics Committee of the Third People’s Hospital of Yunnan Province (approval number: 2023KY128). All procedures were conducted in accordance with the ethical principles outlined in the Declaration of Helsinki. Written informed consent was obtained from all participants prior to enrollment. Participants were informed of the study objectives, intervention protocols, potential risks and benefits, and their right to withdraw at any time without impact on their standard medical care. Patient data were anonymized and handled in compliance with applicable data protection regulations.

### Inclusion criteria

Age ≥18 years, with no restriction on sex.

Confirmed diagnosis of internal hemorrhoids (Grade II or III) by clinical examination and anoscopy.

Scheduled to undergo endoscopic RBL, endoscopic IS, or combined endoscopic RBL and IS treatment.

Normal cognitive function with capacity to cooperate with study assessments.

Voluntary participation with written informed consent.

### Exclusion criteria

Concurrent perianal conditions including anal fissure, anal fistula, or rectal polyps.

Severe cardiac, pulmonary, hepatic, or renal dysfunction.

Coagulation disorders or current anticoagulant therapy.

Known allergy to Kangfuxin solution or materials used in the study.

Poor compliance or inability to cooperate with study procedures.

### Dropout criteria

Failure to implement the intervention protocol as planned.

Voluntary withdrawal from the study for any reason.

Incomplete data precluding statistical analysis.

### Sample size estimation and group allocation

Sample size was estimated using PASS 15.0 software. Based on preliminary data, the anticipated improvement rates for anal fullness symptoms were 85% in the combined intervention group and 60% in the routine care group. With a two-sided *α* of 0.05 and a statistical power of 90% (1 − *β* = 0.90), the minimum required sample size was 26 patients per group. To account for a potential dropout rate of approximately 15%, the sample size was increased to 30 patients per group, yielding a total enrollment of 120 patients. Patients were sequentially allocated to one of the four groups (30 per group) using the sealed envelope method described above, with allocation performed by a research nurse who was not involved in intervention delivery, outcome assessment, or data analysis.

### Intervention protocols

All interventions commenced within 6 h following the endoscopic procedure and continued for 5 consecutive days. All four groups received identical routine postoperative nursing care. Groups A and B additionally received Kangfuxin solution perianal warm moist compress, Groups A and C additionally received music therapy, and Group D received routine care only. All interventions were administered by trained nursing staff who had undergone standardized protocol training prior to study commencement to ensure consistency in intervention quality.

### Kangfuxin solution perianal warm moist compress

Kangfuxin solution (*Periplaneta americana* extract; Yunnan Sainuo Pharmaceutical Co., Ltd., China) was prepared by diluting 100 mL of the solution in 500 mL of warm boiled water, mixed thoroughly, and heated to 40–45 °C. Patients were positioned in the left lateral decubitus position with the hip elevated to fully expose the perianal region. A sterile gauze pad (approximately 30 cm × 30 cm) was completely immersed in the prepared solution, wrung until non-dripping, and applied to the perianal area covering the anus and approximately 10 cm of the surrounding tissue. Each application lasted 20 min, during which the compress was replaced 2–3 times as the temperature decreased. The procedure was performed twice daily (morning and evening) for 5 consecutive days. Nursing staff monitored compress temperature throughout each session, ensuring patient comfort while avoiding thermal injury. The compress was replaced immediately if patients reported any skin discomfort such as pruritus or burning sensation.

### Music therapy

Music therapy sessions were conducted twice daily (morning and evening) for 30 min per session over 5 consecutive days. Selection of music was individualized based on patient preference, with options including classical music, light instrumental compositions, and nature-inspired soundscapes. Purely instrumental tracks were prioritized to minimize cognitive interference from lyrical content. Prior to each session, the ward environment was optimized by adjusting ambient lighting and room temperature to promote relaxation. Portable audio devices (MP3 players or tablet computers) equipped with comfortable headphones were provided, and volume was adjusted to each patient’s preferred comfort level. During sessions, patients were instructed to close their eyes, maintain a relaxed posture, and minimize verbal or physical activity.

### Routine postoperative nursing care

All patients across the four groups received standardized routine postoperative nursing care, which included: continuous monitoring of vital signs and observation for hematochezia, abdominal distension, and abdominal pain; bed rest with avoidance of upright positioning for the initial 24 h postoperatively and restriction of strenuous exercise and heavy physical labor for 2 weeks; dietary progression from a liquid diet on postoperative day 1 to a semi-liquid diet during the first week, with gradual transition to a regular diet while avoiding acidic, spicy, irritating, and coarse foods; maintenance of regular bowel habits with judicious use of osmotic laxatives (e.g., lactulose) as needed; deep vein thrombosis prophylaxis through guided ankle pump exercises and lower extremity compression therapy; and initiation of pelvic floor exercises (levator ani muscle training) and sitz bath therapy beginning 24 h after the procedure. Discharge instructions included alcohol abstinence for 3 months, cultivation of regular bowel habits and dietary practices, and engagement in moderate physical activity to reduce the risk of hemorrhoid recurrence.

### Group-specific intervention summary

Group A received the combined intervention of Kangfuxin solution perianal warm moist compress and music therapy in addition to routine care. Group B received Kangfuxin solution perianal warm moist compress plus routine care. Group C received music therapy plus routine care. Group D received routine postoperative nursing care alone.

### Outcome measures

#### Primary outcome: anal fullness symptom score

The primary outcome was the severity of anal fullness symptoms assessed using the Visual Analogue Scale (VAS), scored on a continuous scale from 0 (no discomfort) to 10 (maximum discomfort) (12). In this study, the VAS was specifically used to assess the subjective intensity of anal fullness, defined as the sensation of rectal pressure, distension, or heaviness in the perianal region. Patients were instructed to rate the severity of the fullness sensation specifically, distinguishing it from pain or other postoperative symptoms. VAS has been widely used to quantify subjective symptom severity in proctology research, including studies assessing postoperative anorectal discomfort and fullness (13, 14). Assessments were performed at baseline (preoperatively) and on postoperative days 1, 3, 5, and 7. Patients independently marked their symptom severity on the VAS ruler, and scores were recorded by the attending nursing staff.

#### Secondary outcomes

Duration of anal fullness symptoms: The symptom duration was defined as the interval from the onset of anal fullness following surgery to complete resolution of symptoms, recorded in days. Patients were assessed daily by nursing staff to document symptom relief until full resolution was confirmed.

Quality of life: Health-related quality of life was evaluated using the World Health Organization Quality of Life–Brief Version (WHOQOL-BREF) instrument (15), encompassing four domains: physical health, psychological wellbeing, social relationships, and environmental satisfaction, comprising a total of 26 items. Each item was scored on a 5-point Likert scale ranging from 1 (very dissatisfied) to 5 (very satisfied), with domain scores calculated as the sum of constituent items; higher scores indicated better quality of life. Assessments were performed preoperatively and on postoperative day 5. Patients completed the questionnaire independently, with nursing staff available to provide clarification on item interpretation. Completed questionnaires were collected and reviewed for completeness on the same day.

Therapeutic efficacy: Clinical efficacy was classified into three categories based on the percentage reduction in VAS score on postoperative day 5 relative to the preoperative baseline: markedly effective (≥75% reduction), effective (≥50% but <75% reduction), and ineffective (<50% reduction). The total effective rate was calculated as the proportion of patients achieving either markedly effective or effective outcomes. These classification thresholds were adapted from established criteria commonly used in traditional Chinese medicine clinical research for symptom-based outcome evaluation and are consistent with published proctology trials employing VAS-based efficacy grading (16).

Adverse events: All adverse events occurring during the 5-day intervention period were prospectively recorded, including local skin reactions (pruritus, erythema, or burning sensation) and any systemic adverse effects. Severity and management of each event were documented.

### Statistical analysis

All statistical analyses were performed using SPSS version 26.0 (IBM Corp., Armonk, NY, USA). Continuous variables were first assessed for normality using the Shapiro–Wilk test and for homogeneity of variance using Levene’s test. Data conforming to a normal distribution were expressed as mean ± standard deviation (x̄ ± s) and compared across groups using one-way analysis of variance (ANOVA), with *post hoc* pairwise comparisons performed using the least significant difference (LSD) t-test. Non-normally distributed data were reported as median with interquartile range [M(Q1, Q3)] and analyzed using the Kruskal–Wallis H test, with pairwise comparisons conducted via the Mann–Whitney U test. Categorical variables were expressed as frequencies and percentages [*n* (%)] and compared using the chi-square (*χ*^2^) test, with Bonferroni correction applied for multiple comparisons where appropriate.

Repeated measures of anal fullness symptom scores were analyzed using generalized estimating equations (GEE) to evaluate the effects of time, intervention group, and their interaction on symptom trajectory. An exchangeable working correlation structure was assumed, and model parameters were estimated using robust standard errors. Kaplan–Meier survival analysis was employed to characterize the time-to-resolution of anal fullness symptoms, with inter-group differences assessed by the log-rank test. Multiple linear regression analysis was performed to identify independent predictors of postoperative quality of life. All tests were two-sided, with a significance level set at *p* < 0.05.

To leverage the 2 × 2 factorial design, two-way analysis of variance was additionally performed to assess the independent main effects of the Kangfuxin solution factor (KFX: present [Groups A + B] vs. absent [Groups C + D]) and the music therapy factor (music: present [Groups A + C] vs. absent [Groups B + D]), as well as their interaction (KFX × music), on the primary outcome (VAS at postoperative day 5) and symptom duration. The interaction term was examined to determine whether the combined effect was additive (non-significant interaction, indicating independent contributions) or synergistic (significant positive interaction, indicating a multiplicative benefit exceeding the sum of individual effects).

## Results

### Baseline characteristics

**Figure 1:**
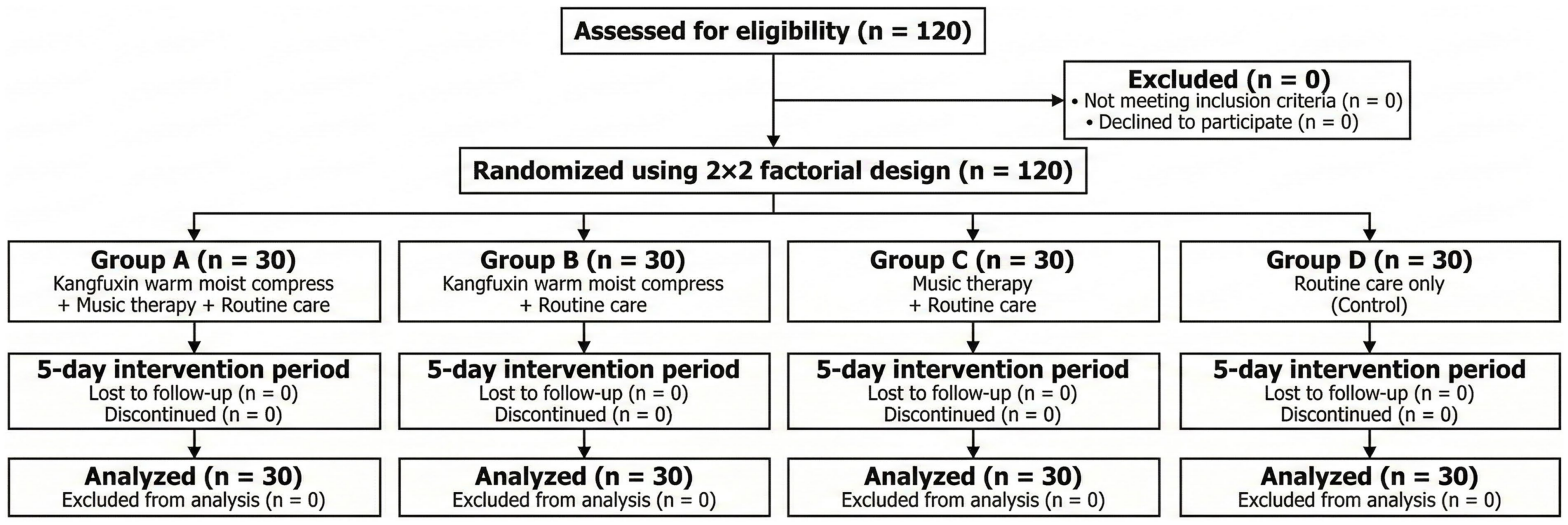
Flow diagram of participant enrollment, allocation, follow-up, and analysis. A total of 120 patients were assessed for eligibility and randomized using a 2 × 2 factorial design into four groups (*n* = 30 per group): Group A (Kangfuxin solution perianal warm moist compress + music therapy + routine care), Group B (Kangfuxin solution perianal warm moist compress + routine care), Group C (music therapy + routine care), and Group D (routine care only). No patients were lost to follow-up or excluded from analysis.

A total of 120 patients meeting the eligibility criteria were enrolled and allocated to the four study groups, with 30 patients in each arm. No patients were lost to follow-up or withdrew during the study period (Figure 1), and all 120 participants completed the full intervention protocol and were included in the final analysis. The baseline demographic and clinical characteristics of the four groups are summarized in Table 1. There were no statistically significant differences among the groups with respect to sex distribution (*χ*^2^ = 0.536, *p* = 0.911), age (*F* = 0.382, *p* = 0.766), body mass index (*F* = 0.297, *p* = 0.828), internal hemorrhoid grade (II vs. III; *χ*^2^ = 0.669, *p* = 0.880), or operative duration (*F* = 0.814, *p* = 0.489), confirming adequate comparability across groups at baseline (Table 1).

**Table 1:** Comparison of baseline demographic and clinical characteristics across the four groups.

Group	*n*	Sex (M/F)	Age (years)	BMI (kg/m^2^)	Hemorrhoid grade (II/III)	Operative duration (min)
Group A	30	17/13	38.5 ± 10.2	23.6 ± 3.5	19/11	28.6 ± 6.2
Group B	30	16/14	40.2 ± 9.8	24.2 ± 3.8	20/10	30.2 ± 7.1
Group C	30	18/12	39.6 ± 10.5	23.9 ± 3.6	18/12	29.5 ± 6.8
Group D	30	15/15	41.3 ± 10.1	24.5 ± 3.7	21/9	31.3 ± 7.4
*χ*^2^/*F*		0.536	0.382	0.297	0.669	0.814
*p* value		0.911	0.766	0.828	0.880	0.489

Values are presented as *n*, n/n, or mean ± standard deviation. BMI, body mass index; M, male; F, female. Hemorrhoid grades based on the Goligher classification.

### Anal fullness symptom scores

Preoperative VAS scores for anal fullness were comparable across the four groups, with no significant inter-group differences at baseline (Table 2). Following surgery, all groups demonstrated a progressive decline in symptom severity over the observation period; however, the magnitude of improvement differed substantially among the intervention arms.

**Table 2:** Comparison of anal fullness Visual Analogue Scale scores (mean ± SD) across the four groups at each time point.

Group	*n*	Preop.	POD 1	POD 3	POD 5	POD 7
Group A	30	7.26 ± 1.52	4.13 ± 1.08*^#†^	2.85 ± 0.92*^#†^	1.74 ± 0.71*^#†^	0.82 ± 0.53*^#†^
Group B	30	7.31 ± 1.49	4.82 ± 1.15*^#^	3.47 ± 1.02*^#^	2.39 ± 0.83*^#^	1.28 ± 0.65*^#^
Group C	30	7.28 ± 1.55	5.56 ± 1.24*	4.21 ± 1.13*	3.02 ± 0.95*	1.85 ± 0.72*
Group D	30	7.33 ± 1.47	6.25 ± 1.31	5.14 ± 1.20	3.87 ± 1.06	2.53 ± 0.88

Preop., preoperative; POD, postoperative day. *p< 0.05 vs. Group D; ^#^*p* < 0.05 vs. Group C; ^†^*p* < 0.05 vs. Group B.

On postoperative day 1, Groups A and B exhibited significantly lower VAS scores than Groups C and D (all *p* < 0.05), with Group A demonstrating the greatest reduction (4.13 ± 1.08). By postoperative day 3, the inter-group separation became more pronounced, with Group A achieving a mean score of 2.85 ± 0.92 compared with 3.47 ± 1.02 in Group B, 4.21 ± 1.13 in Group C, and 5.14 ± 1.20 in Group D. This trend continued through postoperative day 5, when Group A recorded the lowest VAS score (1.74 ± 0.71), significantly surpassing all other groups (all *p* < 0.05). By postoperative day 7, symptom scores in Group A had declined to 0.82 ± 0.53, reflecting near-complete resolution, whereas Group D retained a mean score of 2.53 ± 0.88 (Table 2).

The GEE analysis revealed that time (Wald *χ*^2^ = 1,263.528, *p* < 0.001), intervention method (Wald *χ*^2^ = 98.337, *p* < 0.001), and their interaction (Wald *χ*^2^ = 30.972, *p* < 0.001) all exerted significant effects on anal fullness symptom trajectories, indicating that the rate of symptom improvement differed significantly across intervention groups over time, with Group A demonstrating the most rapid and sustained reduction in anal fullness severity.

### Factorial main effects and interaction analysis

To evaluate the independent contributions of each intervention and their potential interaction, two-way factorial ANOVA was performed on the primary outcome (VAS score at postoperative day 5) and symptom duration.

For VAS scores on postoperative day 5, the Kangfuxin solution factor (KFX: Groups A + B vs. Groups C + D) demonstrated a significant main effect (*F* = 42.15, *p* < 0.001), with patients receiving Kangfuxin compress demonstrating a mean VAS score of 2.07 ± 0.81 compared with 3.45 ± 1.05 in those without. The music therapy factor (Groups A + C vs. Groups B + D) also showed a significant main effect (*F* = 15.83, *p* < 0.001), with mean VAS scores of 2.38 ± 0.97 versus 3.13 ± 1.12. The KFX × music interaction was not statistically significant (*F* = 0.42, *p* = 0.518), indicating that the effects of the two interventions were additive rather than synergistic.

Similarly, for symptom duration, the Kangfuxin solution factor demonstrated a significant main effect (*F* = 52.38, *p* < 0.001; mean 4.31 ± 1.22 vs. 6.57 ± 1.56 days), and the music therapy factor also showed a significant main effect (*F* = 12.76, *p* < 0.001; mean 4.89 ± 1.42 vs. 5.99 ± 1.65 days). The interaction term was again non-significant (*F* = 0.58, *p* = 0.448), confirming the additive nature of the combined benefits (Supplementary Table S1).

### Duration of anal fullness symptoms

The mean duration of anal fullness symptoms differed significantly among the four groups (*χ*^2^ = 58.436, *p* < 0.001). Group A experienced the shortest symptom duration at 3.85 ± 1.12 days, followed by Group B (4.76 ± 1.25 days), Group C (5.92 ± 1.38 days), and Group D (7.21 ± 1.56 days). Pairwise comparisons demonstrated statistically significant differences between all group pairs (all *p* < 0.05), establishing a clear hierarchy of benefit: combined intervention > Kangfuxin solution alone > music therapy alone > routine care.

**Figure 2:**
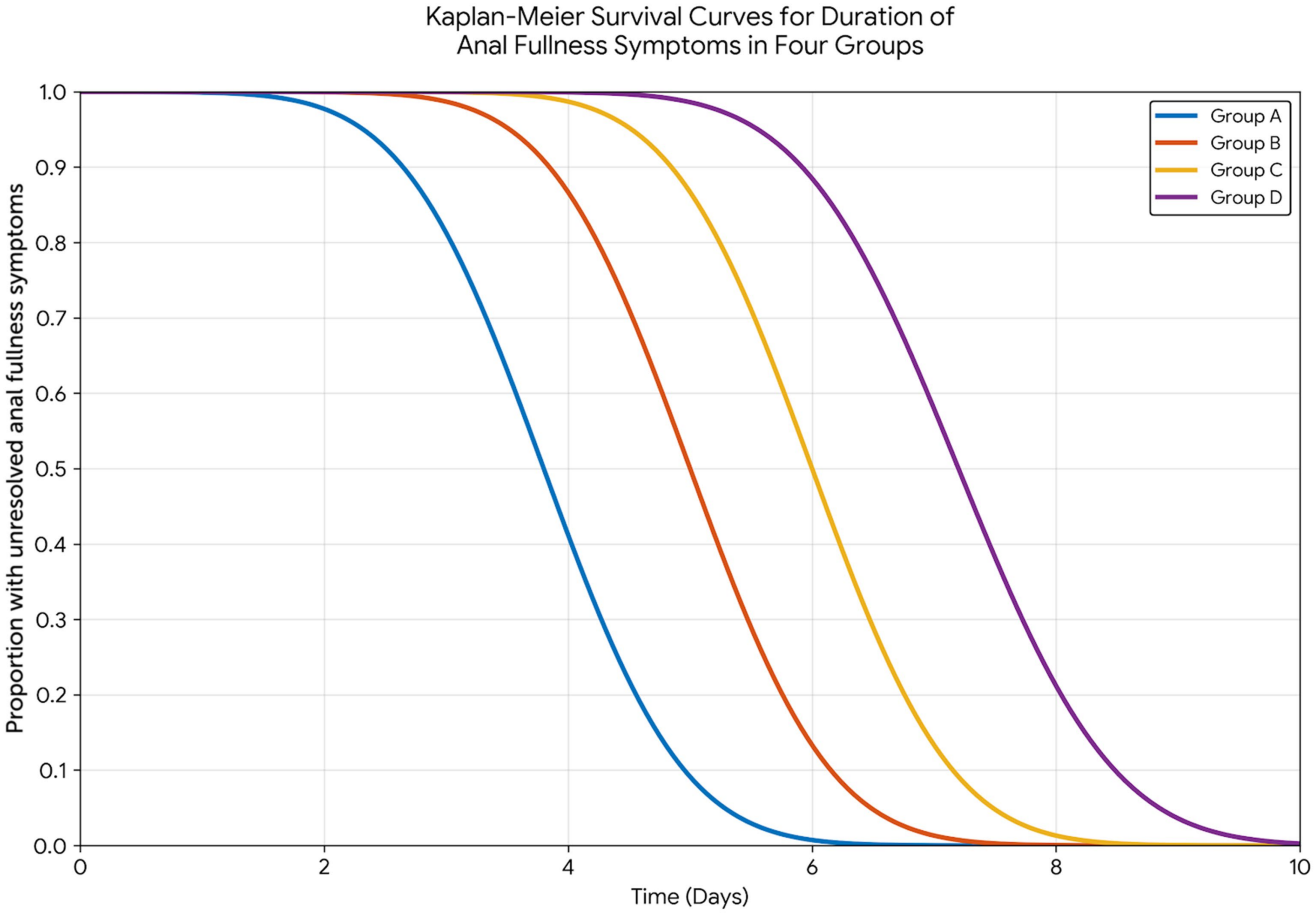
Kaplan–Meier survival analysis of time to resolution of anal fullness symptoms across the four intervention groups. Group A, Kangfuxin solution perianal warm moist compress combined with music therapy; Group B, Kangfuxin solution perianal warm moist compress alone; Group C, music therapy alone; Group D, routine postoperative nursing care. The *x*-axis represents time (days) following surgery, and the *y*-axis represents the proportion of patients with unresolved anal fullness symptoms. Pairwise comparisons by log-rank test demonstrated statistically significant differences between all group pairs (all *p* < 0.05). The combined intervention group (Group A) exhibited the most rapid decline in symptom persistence, with the majority of patients achieving complete symptom resolution within 4–5 days postoperatively.

The Kaplan–Meier survival analysis further illustrated these differences in time-to-symptom resolution, with the survival curves for the four groups showing distinct separation patterns (Figure 2). The log-rank test confirmed statistically significant differences in symptom duration distributions across all groups. Group A exhibited the steepest decline in the proportion of patients with unresolved symptoms, with the majority achieving complete relief within the first 4–5 days, whereas Group D showed a more protracted recovery trajectory.

### Quality of life assessment

Preoperative WHOQOL-BREF domain scores were comparable across the four groups, with no statistically significant baseline differences in the physical, psychological, social relationship, or environmental domains (Table 3). On postoperative day 5, all intervention groups (A, B, and C) demonstrated improvements in quality of life scores relative to baseline, while Group D showed minimal change.

**Table 3:** Comparison of WHOQOL-BREF domain scores (mean ± SD) before and 5 days after surgery across the four groups.

Group	*n*	Time	Physical health	Psychological	Social relationships	Environmental
Group A	30	Preop.	12.26 ± 2.14	11.85 ± 2.06	10.92 ± 1.88	11.53 ± 2.02
		POD 5	16.53 ± 2.31*^#†^	15.82 ± 2.15*^#†^	14.76 ± 2.03*^#†^	15.24 ± 2.12*^#†^
Group B	30	Preop.	12.31 ± 2.18	11.92 ± 2.11	10.88 ± 1.92	11.47 ± 2.06
		POD 5	15.14 ± 2.25*^#^	14.35 ± 2.08*^#^	13.29 ± 1.96*^#^	14.02 ± 2.05*^#^
Group C	30	Preop.	12.28 ± 2.20	11.89 ± 2.09	10.94 ± 1.90	11.56 ± 2.08
		POD 5	13.82 ± 2.16*	13.11 ± 2.01*	12.08 ± 1.89*	12.85 ± 1.98*
Group D	30	Preop.	12.33 ± 2.17	11.95 ± 2.13	10.91 ± 1.93	11.50 ± 2.04
		POD 5	12.45 ± 2.12	12.02 ± 2.05	11.03 ± 1.87	11.62 ± 1.99

WHOQOL-BREF, World Health Organization Quality of Life–Brief Version; Preop., preoperative; POD, postoperative day. *p < 0.05 vs. Group D; ^#^*p* < 0.05 vs. Group C; ^†^*p* < 0.05 vs. Group B.

Group A achieved the highest postoperative scores across all four WHOQOL-BREF domains: physical health (16.53 ± 2.31), psychological wellbeing (15.82 ± 2.15), social relationships (14.76 ± 2.03), and environmental satisfaction (15.24 ± 2.12), all of which were significantly superior to Groups B, C, and D (all *p* < 0.05). Group B demonstrated significantly higher scores than Group C across all domains, while Group C achieved significantly higher scores than Group D (Table 3). The physical health domain exhibited the most pronounced inter-group differences, consistent with the direct symptomatic benefits of the interventions on postoperative anal fullness.

Multiple linear regression analysis identified the intervention method as the predominant independent predictor of postoperative quality of life (*β* = 0.682, *p* < 0.001), followed by age (*β* = −0.214, *p* = 0.006) and internal hemorrhoid grade (*β* = −0.187, *p* = 0.015). These results suggest that the choice of nursing intervention had the greatest influence on postoperative quality of life, while older age and higher hemorrhoid grade were independently associated with poorer outcomes.

### Therapeutic efficacy

The overall distribution of therapeutic efficacy differed significantly among the four groups (*χ*^2^ = 11.196, *p* = 0.011). Group A achieved the highest total effective rate of 96.67% (24 markedly effective, 5 effective, 1 ineffective), followed by Group B at 93.33% (20 markedly effective, 8 effective, 2 ineffective), Group C at 83.33% (15 markedly effective, 10 effective, 5 ineffective), and Group D at 70.00% (9 markedly effective, 12 effective, 9 ineffective). Notably, the proportion of patients achieving markedly effective outcomes was higher in the groups receiving Kangfuxin solution (Groups A and B) compared with those who did not (Groups C and D), underscoring the contribution of the topical intervention to clinical outcomes.

### Adverse events

No serious adverse events were observed in any of the four groups during the study period. In Group A, one patient (3.33%) reported mild local skin pruritus at the compress application site, which resolved promptly following symptomatic management without interruption of the intervention protocol. In Group B, two patients (6.67%) experienced similar mild local pruritus, both of which were successfully managed with symptomatic treatment. No adverse events were reported in Group C or Group D. All reported adverse events were classified as mild in severity, self-limiting in nature, and did not necessitate discontinuation of the study intervention. The overall incidence of adverse events was 2.5% (3/120), supporting the favorable safety profile of both interventions.

## Discussion

This study evaluated the efficacy of Kangfuxin solution perianal warm moist compress combined with music therapy for the management of anal fullness following endoscopic treatment of internal hemorrhoids. The results demonstrate that this combined nursing intervention significantly alleviates postoperative anal fullness symptoms, shortens symptom duration, and improves quality of life compared with either intervention alone or routine care.

The combined intervention group (Group A) exhibited consistently lower VAS scores at all postoperative time points and the shortest symptom duration among the four groups, with GEE analysis confirming a significant time-by-intervention interaction effect. These findings suggest that the concurrent application of Kangfuxin solution warm moist compress and music therapy produces complementary benefits that exceed the effects of either modality in isolation. Notably, the formal two-way factorial analysis revealed that the interaction between the two interventions was not statistically significant, indicating that their combined benefit is additive rather than synergistic. In other words, each intervention contributes independently to symptom relief, and the superiority of the combined approach reflects the cumulative effect of two independent therapeutic mechanisms rather than a multiplicative interaction. The Kangfuxin solution factor demonstrated a larger main effect than the music therapy factor on both VAS scores and symptom duration, consistent with the group-level comparisons showing greater benefit from the topical intervention. This additive pattern is clinically meaningful because it supports the rationale for combining the two interventions: each contributes a distinct and independent layer of benefit, and their joint use maximizes overall therapeutic effect without requiring a synergistic mechanism.

Kangfuxin solution is an established traditional Chinese medicine preparation derived from the extract of *Periplaneta americana*, with pharmacological properties including promotion of tissue repair, anti-inflammatory activity, and enhancement of local microcirculation (17). When applied as a perianal warm moist compress, the solution enables direct topical delivery of its bioactive components to the perianal tissues, while the thermal effect of the compress promotes vasodilation, increases local blood flow, and facilitates tissue healing (18, 19).

Music therapy contributes to symptom relief through a distinct mechanism. By providing structured auditory stimulation through carefully selected melodies and rhythms, music therapy redirects patient attention, promotes psychophysiological relaxation, and modulates pain perception through descending inhibitory pathways (20, 21). Palakanis et al. (22) demonstrated that music therapy significantly reduced state anxiety in patients undergoing colorectal procedures, supporting its role as an effective complementary nursing intervention in this clinical context. The additive effect observed in Group A likely reflects the convergence of these two complementary mechanisms: the peripheral pharmacological and thermal effects of Kangfuxin solution address local tissue inflammation and circulation, while music therapy modulates the central pain-processing and stress-response systems.

Both the Kangfuxin solution group (Group B) and the music therapy group (Group C) demonstrated significant improvements in anal fullness symptoms and quality of life compared with routine care (Group D), confirming the independent therapeutic value of each intervention. However, the factorial design of this study revealed a clear hierarchy of efficacy, with the combined intervention consistently outperforming either single modality. This pattern was observed across all outcome measures, including VAS scores, symptom duration, WHOQOL-BREF domain scores, and total effective rate.

Interestingly, Kangfuxin solution warm moist compress (Group B) demonstrated greater efficacy than music therapy alone (Group C) across most outcome measures, suggesting that the local pharmacological and physical effects of the topical intervention may provide more direct and substantial symptom relief for anal fullness than the centrally mediated effects of music therapy. Nevertheless, the additional benefit conferred by music therapy when combined with Kangfuxin solution (Group A vs. Group B) was statistically significant, reinforcing the value of multimodal nursing approaches in postoperative care.

Quality of life is an important indicator for evaluating postoperative care quality. The WHOQOL-BREF assessment revealed that all three intervention groups demonstrated significant improvements across the physical, psychological, social relationship, and environmental domains compared with routine care on postoperative day 5. Multiple linear regression analysis identified the intervention method as the strongest independent predictor of postoperative quality of life (*β* = 0.682), indicating that optimizing nursing interventions can meaningfully improve patient outcomes beyond the effects of age and disease severity.

Internal hemorrhoids and their surgical treatment can cause not only physical discomfort but also psychological distress, including anxiety and reduced social functioning (23). The combined intervention addresses both dimensions: Kangfuxin solution warm moist compress targets the local physical symptoms, while music therapy improves emotional wellbeing and promotes a sense of relaxation and comfort. This dual mechanism may account for the comprehensive quality of life improvement observed in Group A across all four WHOQOL-BREF domains.

The safety findings of this study are reassuring. No serious adverse events were observed in any group, and the overall adverse event incidence was low (2.5%). The only reported events were mild local skin pruritus in three patients receiving Kangfuxin solution compress (Groups A and B), all of which resolved spontaneously following symptomatic treatment without requiring intervention discontinuation. These findings are consistent with the established safety profile of Kangfuxin solution for external application, for which adverse reactions are reported to be rare and generally limited to mild, transient local skin reactions (8, 24). Music therapy, as a non-pharmacological and non-invasive modality, demonstrated an excellent safety profile with no adverse events reported (25, 26).

Several limitations of this study should be acknowledged. First, this was a single-center study with a relatively small sample size (*n* = 30 per group), which may limit the generalizability of the findings to other clinical settings and patient populations; the relatively small per-group sample size may also have limited statistical power to detect smaller effect sizes, particularly for the factorial interaction analysis. Second, the follow-up period was confined to the immediate postoperative period (5–7 days), and the long-term effects of the combined intervention on hemorrhoid recurrence and sustained quality of life improvement remain unknown. Third, due to the nature of the interventions, blinding of patients and nursing staff to group allocation was not feasible, which may have introduced performance and detection bias. The absence of blinding is particularly relevant given that the primary outcome (VAS) is a subjective self-reported measure, and patients’ awareness of their group assignment may have influenced symptom reporting through expectation effects or placebo response. Fourth, the WHOQOL-BREF assessment was performed on postoperative day 5, which may be too early to capture the full impact of the interventions on quality of life; future studies should consider additional assessments at later time points (e.g., 4–6 weeks postoperatively) to evaluate sustained quality of life benefits. Fifth, this study did not include an objective measure of perianal tissue inflammation or healing (e.g., photographic assessment, perianal edema scoring, or anorectal manometry), which could have provided additional mechanistic insight and strengthened the evidence base beyond subjective patient-reported outcomes. Future studies employing larger, multicenter cohorts with extended follow-up periods, objective outcome measures, and strategies to mitigate bias in unblinded designs (such as blinded outcome assessment by independent evaluators) are warranted to further validate these findings and elucidate the long-term benefits of this combined nursing intervention.

## Conclusion

Kangfuxin solution perianal warm moist compress combined with music therapy effectively alleviates anal fullness following endoscopic treatment of internal hemorrhoids, shortens symptom duration, improves postoperative quality of life, and demonstrates a favorable safety profile. Factorial analysis confirmed that the two interventions exert additive rather than synergistic effects, with each modality contributing independently to improved outcomes. The combined intervention outperforms either modality alone and routine care across all outcome measures. These findings support the integration of this multimodal nursing approach into standardized postoperative care protocols for patients undergoing endoscopic treatment of internal hemorrhoids. Further multicenter studies with larger samples, longer follow-up periods (including assessments at 4–6 weeks and beyond), objective outcome measures, and blinded outcome assessment are needed to confirm these results and evaluate long-term efficacy.

## Data Availability

The original contributions presented in the study are included in the article/Supplementary material, further inquiries can be directed to the corresponding author.
