# Supplementary material for: Kangfuxin solution perianal warm moist compress combined with music therapy for anal fullness after endoscopic treatment of internal hemorrhoids
Source: Front Med (Lausanne). 2026 May 11;13:1835242. doi: 10.3389/fmed.2026.1835242 (PMC13199331; doi:10.3389/fmed.2026.1835242)
Supplement: Supplementary file 1 [file Table_1.DOCX]

**Supplementary Table S1. Two-way factorial analysis of main effects and interaction for primary and secondary outcomes.**

| **Outcome** | **Factor** | **Groups** | **Mean ± SD** | **F** | **P value** |
| --- | --- | --- | --- | --- | --- |
| VAS (POD 5) | KFX (present) | A + B (n = 60) | 2.07 ± 0.81 | 42.15 | < 0.001 |
|  | KFX (absent) | C + D (n = 60) | 3.45 ± 1.05 |  |  |
|  | Music (present) | A + C (n = 60) | 2.38 ± 0.97 | 15.83 | < 0.001 |
|  | Music (absent) | B + D (n = 60) | 3.13 ± 1.12 |  |  |
|  | KFX × Music | — | — | 0.42 | 0.518 |
| Symptom duration (days) | KFX (present) | A + B (n = 60) | 4.31 ± 1.22 | 52.38 | < 0.001 |
|  | KFX (absent) | C + D (n = 60) | 6.57 ± 1.56 |  |  |
|  | Music (present) | A + C (n = 60) | 4.89 ± 1.42 | 12.76 | < 0.001 |
|  | Music (absent) | B + D (n = 60) | 5.99 ± 1.65 |  |  |
|  | KFX × Music | — | — | 0.58 | 0.448 |

*Mean values for pooled groups are calculated directly from original group means. VAS, Visual Analogue Scale; POD, postoperative day; KFX, Kangfuxin solution factor.*
